# Multicultural Toronto and the Building of an Ethnic Landscape: Chronic Urban Trauma

**DOI:** 10.3390/bs16020175

**Published:** 2026-01-26

**Authors:** Carlos Teixeira

**Affiliations:** Geography/Community, Culture and Global Studies, University of British Columbia, Kelowna, BC V1V 1V7, Canada; carlos.teixeira@ubc.ca

**Keywords:** immigrant youth trauma, chronic urban trauma, Portuguese-Azorean diaspora, ethnic enclaves, multiculturalism, gentrification

## Abstract

This paper investigates how Toronto’s Portuguese-Azorean community has shaped the city’s multicultural and psychological landscape, focusing particularly on intergenerational experiences of trauma among immigrant youth. Framed within North America’s broader migration dynamics, the study explores the creation and transformation of the ethnic enclave “Little Portugal” as both a space of cultural resilience and chronic urban stress. It introduces the concept of chronic urban trauma to describe the persistent psychosocial impact of displacement, assimilation pressures, and gentrification on young Portuguese-Azorean Canadians. While first-generation immigrants constructed cohesive ethnic infrastructures grounded in work, faith, and language, younger generations face cultural dissonance, linguistic loss, and identity fragmentation that manifest as emotional distress and social alienation. These experiences illustrate how structural urban change can perpetuate transgenerational trauma within immigrant families. By integrating perspectives from urban geography, trauma studies, and migration theory, this theoretical work underscores the need for trauma-informed educational and social policies that promote inclusion, belonging, and mental well-being among immigrant youth. Ultimately, the study positions “Little Portugal” as a microcosm of how multicultural cities negotiate the intersections of ethnicity, urban transformation, and psychological resilience.

## 1. Introduction

Since the end of the Second World War, significant changes in immigration policies and global migration patterns have transformed major North American metropolitan areas into multicultural gateway cities. In the contemporary “age of migration,” cities such as Los Angeles, New York, Miami, Toronto, Vancouver, and Montreal have become primary destinations, reshaping the socio-demographic composition of urban neighbourhoods (Murdie & Teixeira, 2015; Szkudlarek & Wu, 2018; Yasin & Hafeez, 2023). A common characteristic of recent immigrants in both countries is their preference for settling in major urban areas, with the majority of immigrants in Canada and the United States residing in metropolitan contexts (Drolet & Teixeira, 2020; Kobayashi et al., 2012).

While this “diversity explosion” has enriched North American societies economically and culturally, immigrant settlement is also accompanied by structural challenges related to housing access, labour market integration, social exclusion, and neighbourhood transformation. Refugees and working-class immigrants, in particular, often experience layered forms of vulnerability associated with forced displacement, socioeconomic precarity, and post-migration stress (Figueiredo et al., 2024; Brooks et al., 2024). These pressures extend beyond individual experiences, affecting family networks, community cohesion, and intergenerational well-being.

In this context, this study adopts the concept of chronic urban trauma as its central analytical framework to capture how prolonged exposure to spatial displacement, assimilation pressures, gentrification, and institutional marginalization within metropolitan environments shapes immigrant experiences across generations. Trauma in urban immigrant settings encompasses not only psychological distress, but also the erosion of social networks, cultural institutions, and neighbourhood-based support systems that historically anchored everyday life. Such cumulative processes can generate enduring patterns of alienation, cultural dislocation, and emotional strain, particularly among immigrant youth.

At the same time, immigrants continue to contribute substantially to urban economic development, demographic renewal, and cultural diversification. Their participation in labour markets, entrepreneurship, and transnational economic networks has become increasingly important in post-industrial urban economies (Banerjee et al., 2021; Ley, 2010; Sithas & Surangi, 2021). However, public and political debates regarding immigration levels, settlement distribution, and integration policies persist, reflecting ongoing tensions between economic dependence on migration and uneven social incorporation (Banyard et al., 2025; Sweet & Harper-Anderson, 2023). In this discourse we find other issues: educational strategies to enable inclusion for young immigrants, especially in schools of Canada (Este & Ngo, 2011; Tardif-Grenier et al., 2023; Weisleder et al., 2024).

**Figure 1:**
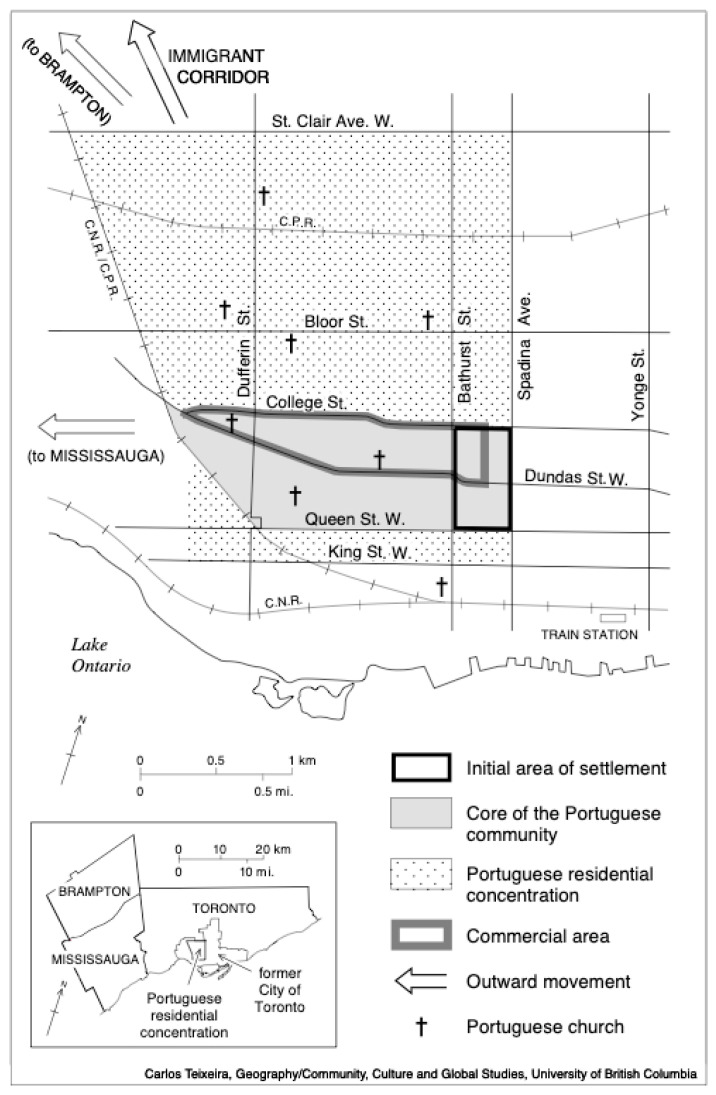
Portuguese immigrants’ generations in Toronto areas.

Given ageing populations, immigration’s demographic importance to the U.S. and Canada is clear. Today’s North American cities are characterized by a mix of cultural and immigrant groups settled in a diverse array of ethnic neighbourhoods and diasporic communities (Banyard et al., 2025). Children migrating both from less developed countries and from Western nations contribute to reshaping Canadian society, particularly through the introduction of new skills and professions once their education is completed (Banyard et al., 2025; Parameswaran et al., 2024). Despite the numerous challenges they face, these immigrants are shaping the physical, social, and economic landscapes of major North American cities, states, and provinces in important ways (Sweet & Harper-Anderson, 2023). Scholars predict that neither the United States nor Canada will have a majority ethnic group by mid-century, raising questions about how ethnic identity shapes urban social geography (Dixon et al., 2022; Kobayashi et al., 2012; Preston et al., 2022; U.S. Census Bureau, 2024). Evolving immigrant settlement patterns are transforming the socio-economic and cultural fabric of cities and suburbs, raising critical questions about policy, identity, and belonging (Chen et al., 2023; Kobayashi et al., 2012; Sweet & Harper-Anderson, 2023). Toronto’s well-established Portuguese-Azorean immigrant enclave—“Little Portugal,” or the “Azorean Tenth Island”—offers a key social–cultural laboratory in which to study the role and impact of these immigrants on the urban and social/cultural, political and economic landscapes, and for the study of multiculturalism in one of the most ethnically diverse cities in the world (Figure 1). Ethnic enclaves have become increasingly important elements of Toronto’s urban landscape. Over time, the Portuguese-Azorean community has incorporated ethnic businesses, social, cultural and religious institutions, gardens and shrines, architectural features, cultural symbols as well as other distinguishing physical–cultural characteristics. Studying ethnic enclaves can help us better understand how immigrant groups transform the built environment and integrate into the social–cultural and economic life of a new society. On the other hand, new developmental challenges and characteristics are being observed among immigrant children—phenomena that were not previously documented. Canada has long been regarded as a model for inclusive policies aimed at supporting traumatized children from immigrant backgrounds, particularly when socioeconomic conditions are taken into account (Walls, 2023). Children from the Azorean Islands, for instance, have often been associated with socioeconomic deprivation affecting their families as a whole. However, this population remains understudied in terms of its academic and socioemotional development within contemporary Canadian society and other societies with high density urban areas with immigration settled (Chen et al., 2023).

This theoretical study explores the emergence of “Little Portugal” within Toronto’s urban social mosaic, with a specific focus on the settlement and housing experiences, residential patterns and integration of Portuguese-Azoreans and their descendants born in Canada. It also explores issues related to gentrification as chronic urban trauma, with a particular focus on Portuguese seniors—the first-generation immigrant population who built the community—and Portuguese youth facing pressures of assimilation and displacement. This paper is based on secondary data synthesis, prior empirical studies by the author (see Murdie & Teixeira, 2006, 2015; A. Oliveira & Teixeira, 2004; Teixeira, 2009, 2010; Vieira & Teixeira, 2023). In May 2023, the author revisited the study area and conducted additional fieldwork in the City of Toronto and its suburbs. Notably, this period coincided with commemorations marking the 70th anniversary of the arrival of the first Portuguese “pioneers” in Canada (May 1953). Within this context, the author was invited to present a paper—“*A Presença Portuguesa no Canadá: Uma Perspetiva de Sete Décadas/The Portuguese Presence in Canada: A Perspective of Seven Decades*”—in Montreal (House of the Azores) and Toronto (Brampton Convention Centre), along with the presentation of a book dedicated to the Portuguese in Canada, for which the author wrote the preface. These events were followed by interviews with local Portuguese-language media, including television and print outlets. Participation in these socio-cultural events provided valuable opportunities to reconnect with long-standing and new Portuguese and non-Portuguese contacts in Toronto and Montreal, and, where possible, to engage in informal discussions about the current state and future prospects of Portuguese communities in both cities and across Canada.

### 1.1. The Changing Geography of Ethnicity in North American Cities

U.S. scholars have long modelled North American immigration in the 19th and early 20th centuries as a unidirectional process: migrants left their homelands to recreate familiar cultural settings that eased adjustment. Urban–rural settlement distinctions are recognized as an important dimension in migration research; however, this paper prioritizes urban contexts due to its focus on metropolitan gateway dynamics, enclave formation, and processes of chronic urban trauma. Today’s immigrants—from Asia, Africa, the Middle East, and Latin America—are far more heterogeneous than earlier European arrivals, and their settlement patterns demand renewed attention. Especially considering young immigrants and their maladjustments to schools and to the new routines into the main community (Parameswaran et al., 2024; Zhuang & Lok, 2023). Earlier European immigrants typically settled in inner-city reception areas such as Toronto’s Ward or Montreal’s St. Louis neighbourhood, forming “institutionally complete” enclaves of businesses, churches, and social organizations that reproduced old-world traditions. Over time, many moved to the suburbs in pursuit of homeownership and upward mobility (Drolet & Teixeira, 2020; Chen et al., 2023; Murdie & Teixeira, 2006).

Since the 1970s, however, newer immigrants have often settled directly in suburban areas, bypassing the inner city. In places like Richmond, B.C., and Markham, Ontario, Chinese immigrants from Hong Kong exemplify the creation of “ethnoburbs” (Kobayashi et al., 2012; Preston et al., 2022). These complex patterns challenge traditional spatial assimilation models, suggesting that suburban immigrant settlement reflects a more varied set of economic and cultural choices (Hiebert, 2000; Zhuang & Lok, 2023). The diversity of experiences makes it difficult to identify universal factors behind ethnic and racial segregation (Preston et al., 2022; Zhuang & Lok, 2023). Some low-income immigrants remain concentrated in poor neighbourhoods, while more affluent groups have greater residential flexibility. Across the U.S. and Canada, diverse settlement forms including “ethnic enclaves,” “ethnoburbs,” “invisiburbs,” and “heterolocal communities” underscore the need for clearer frameworks explaining immigrant reception and integration (Skop & Li, 2003; Vine, 2024). Key questions remain about who assimilates whom, who defines the mainstream, and how power and identity are negotiated in multicultural societies. In the middle the traumatized children were forgotten considering the ethnoburbs and mainly the factor of displacement (McFarlane & Hinton, 2009; Pilipossian, 2023). The psychological stress response to these multicultural societies, during its construction, was neglected and converted in chronic urban trauma (Eissa & Zeira, 2024; Hall et al., 2023; Stamm & Stamm, 2013; Vine, 2024).

While many immigrants maintain transnational ties, their children often acculturate quickly through education and media, and intermarriage produces increasingly multiethnic populations. These shifts are redefining what it means to be “North American,” yet they are also creating new social and economic tensions that may reinforce class and ethnic divisions (Ley, 2010; Zhuang & Lok, 2023). Immigration continues to shape the social, cultural, and economic structures of North American cities, but comparative research between the U.S. and Canada remains limited. Both countries face similar challenges: discrimination, economic and housing barriers, and diverging policy responses. Understanding how ethnicity and race influence immigrant integration is therefore central to future studies. Ethnic geographers must examine how enclaves and urban ethnic landscapes evolve under these forces. The case of Portuguese-Azorean immigrants in Toronto’s “Little Portugal,” for example, offers a vivid setting for exploring multiculturalism in one of the world’s most diverse cities.

### 1.2. Chronic Urban Trauma: About the Concept

Chronic urban trauma refers to the cumulative psychological, emotional, and physiological stress experienced by individuals and communities living in urban environments marked by persistent social, economic, and environmental adversity. Unlike acute trauma, which results from discrete events (such as natural disasters, violent incidents, or sudden loss), chronic urban trauma develops gradually through continuous exposure to structural stressors. Its effects are pervasive and long-lasting, shaping mental health, physical well-being, and social functioning over time (Vine, 2024). Recognizing trauma as structurally produced highlights that urban environments themselves can generate persistent stress, rather than trauma being solely a result of individual experiences.

It is important to distinguish chronic urban trauma from related but distinct phenomena. Intergenerational trauma refers to trauma transmitted across generations through familial, social, or epigenetic mechanisms. While chronic urban trauma may contribute to intergenerational stress patterns, it is primarily situated in present-day urban conditions rather than historical or familial legacies. Cultural stress involves pressures to maintain, negotiate, or adapt cultural identity under marginalization, whereas acculturative stress denotes the strain of adapting to a dominant culture, including language barriers and social norms. In urban contexts, acculturative stress can represent one facet of broader chronic trauma, particularly among immigrant or minority populations (Parameswaran et al., 2024).

Empirical observations illustrate how urban processes function as mechanisms of chronic trauma. Housing markets that produce segregation, overcrowding, and displacement generate persistent instability, disrupt social networks, and create ongoing anxiety. Educational pressures, including underfunded schools, overcrowded classrooms, and biassed disciplinary practices, expose students and families to continuous stress, linking structural inequities directly to psychosocial outcomes. Language loss and marginalization in public institutions, media, and services erode cultural identity and social belonging, compounding stress and limiting access to supportive networks. Other urban stressors—such as environmental hazards, policing practices, and economic precarity—similarly operate as structural determinants of trauma, affecting both individual experiences and collective community vulnerability (Chen et al., 2023; Murdie & Skop, 2011). By connecting these empirical examples directly to the framework of chronic trauma, the argument emphasizes that trauma in urban settings is systemically produced rather than episodic or purely personal.

The concept of chronic urban trauma advances urban studies and public health by providing a lens to understand how persistent, structurally embedded stress shapes urban life. Its contribution lies in reframing trauma as a product of urban processes, making visible the often-overlooked ways cities themselves can generate long-term harm. This perspective has practical implications: housing policies should prioritize stability, affordability, and social cohesion to reduce displacement; education systems require targeted support to address inequities and promote culturally responsive, safe learning environments; and language and cultural inclusion programmes can foster belonging and reduce identity-related stress. More broadly, interventions addressing policing, economic precarity, and environmental justice must be designed with awareness of their potential to alleviate or exacerbate chronic trauma.

By linking structural urban conditions directly to psychosocial outcomes, chronic urban trauma offers both a conceptual framework and a roadmap for systemic intervention, encouraging policies and community programmes that target the root causes of urban stress and promote more equitable, resilient cities.

## 2. Portuguese-Azoreans in Multicultural Toronto: The Building and Maintenance of an Urban Ethnic Enclave and What the Future Holds…

### 2.1. Trans-Atlantic Migration and Settlement Patterns

Although large-scale Portuguese and Azorean immigration to Canada officially began in 1953, maritime contact dates back to the late 15th century, when Portuguese navigators fished off Newfoundland and later maintained ties through the “White Fleet” (Collins, 2000; Doel, 1992). Centuries of out-migration from Portugal and the Azores—driven by economic hardship—eventually directed many to Canada in search of work and family reunification (Pires et al., 2023). The first postwar wave of about 17,000 male labourers in the 1950s took up agricultural and railway jobs, followed by chain migration through the 1960s and 1970s. Most settled in Ontario, particularly Toronto, which remains the principal “port of entry.” Today, roughly 450,000 Canadians claim Portuguese origin—two-thirds Azorean—with concentrations in Ontario, Quebec, and British Columbia (Statistics Canada, 2022, 2024).

In the 1950s and 1960s, Portuguese and Azorean immigrants clustered in low-income downtown Toronto neighbourhoods such as Kensington Market, close to industrial work and transit (Teixeira, 2010; Taplin-Kaguru, 2021; Vaz, 2025). Chain migration and shared housing supported high rates of homeownership and strong community networks, making the group among Toronto’s most residentially concentrated (Teixeira & Murdie, 2009). By the mid-1960s, the community had transformed Kensington into a “Portuguese market,” with ethnic businesses, religious clubs, and homes adorned with azulejos and grapevines that recreated rural esthetics (Teixeira, 2009). Homeownership symbolized stability and mobility; many renovated ageing houses with family labour and construction skills, often adding rental suites and cellars (Rivera, 2023; Taplin-Kaguru, 2021).

However, out-migration, limited new arrivals, gentrification, and rising housing costs have weakened Little Portugal’s institutional base and altered its demographic character as occurred already in other main destination of Portuguese immigrants (Marques, 2019; Murdie & Teixeira, 2015). Although many Portuguese-Azoreans now live in the suburbs, the enclave endures symbolically as the “10th Azorean Island,” thus sustaining cultural identity amid ongoing urban transformation (Gendron, 2024; Meligrana & Skaburskis, 2005).

### 2.2. Portuguese and Azorean Seniors in Toronto’s Little Portugal: Resisting Gentrification and Displacement?

Gentrification has been defined as “the loss of affordable older inner-city housing through their renovation and upgrade by middle and upper-income households” (Meligrana & Skaburskis, 2005, p. 1571). Within this context, will gentrification mean the displacement of lower-income households, including the ageing first-generation Portuguese-Azoreans, or are there viable strategies of resistance? Portuguese generally view Little Portugal as a neighbourhood in transition, with gentrification—rising housing prices and rents—affecting both Portuguese (especially seniors) and non-Portuguese residents. Research shows that there are three main groups leaving the area for reasons both voluntary and forced (Takahashi, 2023; Teixeira, 2010). The first are Portuguese and Azoreans in their 40s or 50s who are currently homeowners in the neighbourhood. This is a group with assets and financial stability that aspires to move to the suburbs to improve their housing conditions. The second group are well-off Portuguese and Azorean seniors who, with their mortgages paid, are moving to join children already established in the suburbs (Gendron, 2024; Takahashi, 2023). The third are Portuguese and Azorean seniors who are retired on fixed incomes. Within the first group, we mainly observe children of the second generation in Canada, who experience forms of displacement already encountered by their families, particularly their parents (Vieira & Teixeira, 2023). When reflecting on the Canadian landscape shaped by Portuguese and other immigrant communities, it becomes evident that children remain largely overlooked in scientific research, despite being immigrants who often struggle with social exclusion and learning difficulties. Though Portuguese and Azorean homeowners can profit from Toronto’s rising real estate market, it is clear that, even in the best-case scenario, many Portuguese and Azorean seniors and their children approach and share the prospect of moving with mixed feelings (Murdie & Teixeira, 2015; Takahashi, 2023; Teixeira, 2010).

Despite higher education, occupational skills, and incomes, most Canadian-born Portuguese cannot afford to buy homes in Little Portugal and instead seek affordable housing—either to rent or purchase—in the city’s outskirts and suburbs. To save money, younger generations sometimes rent from or live with their parents in intergenerational households, a practice that has grown since the early 2000s as Toronto’s housing market has become increasingly unaffordable. This daily habit of saving and living under financial constraint has become an internalized behaviour and, for many immigrant children, a source of enduring psychological stress throughout their lives.

While the problem of an ageing Portuguese and Azorean community, particularly its first-generation seniors, has to date received little attention from Canadian scholars and policymakers, it is clear that this group’s cultural needs and preferences with regard to issues of healthcare and wellbeing, ethnically oriented social services (preferably in Portuguese), as well as affordable housing, including long-term care deserves more detailed attention in future (Gomes, 2023; Sampaio, 2022; Takahashi, 2023). On the other hand, the well-being of second-generation immigrant children has received relatively little attention in research to date, even in other contexts where Portuguese populations are significantly represented as immigrant communities (Albert, 2021; Botelho, 2021).

## 3. A Community on the Move: From Voluntary Segregation to Integration

Portuguese and Azorean immigrants have largely integrated into Canadian society, contributing socially, culturally, politically, and economically. First-generation manual labourers were known for their work ethic and thrift, often taking multiple jobs to achieve homeownership. Women balanced full-time work with homemaking, though newer generations are seeing shifts in gender roles.

With regard to cultural traditions, many Portuguese purchase homes as an investment strategy, and their homeownership levels are higher than the Toronto average (Murdie & Teixeira, 2006). It was common for several members of one family to hold more than one job in order to save money to attain the “dream” of owning a home on Canadian soil. Within this context, socio-economic pressures, such the desire to own a house, forced Portuguese and Azorean women to enter the paid workforce, effectively leaving these women with two jobs: full-time worker and traditional homemaker. This excessive burden has frequently affected women’s health. First-generation women’s emancipation has been gradual, but changes in gender roles may reconfigure the Portuguese and Azorean family (Teixeira & Murdie, 2009), and important gains have been made with this regard among the second- and third-generation Portuguese-Azorean women in Canada. Also, for economic reasons, such as buying a house or paying debts in Portugal, many parents forced their children to leave school as soon as possible to supplement the family income. This chronic urban trauma affects the family as a group. We cannot address here the traditional norm of trauma for one individual, but for the group composed by the family and generations of immigrants. Today, 68.4% of Portuguese in Toronto are homeowners, and first-generation residents make up 52.8%. With declining immigration from Portugal, the population is ageing. Latter arrivals—better educated and more skilled—moved beyond the traditional enclave, settling directly further north along the “immigrant corridor” or in Mississauga, and other surrounding suburban municipalities. At this stage little is known about how they relate to the older generations’ cultural heritage, including their housing behaviour and integration.

Family remains a key cultural value among Portuguese-Azorean immigrants, although generational changes are reshaping traditional structures. First-generation families have tended to emphasize large households, extended family ties, and religious values, while younger generations tend to forming smaller households and having fewer children. Over time, financial constraints and cultural assimilation may reduce the ability of younger generations to care for ageing family members, leading to increased reliance on social services and government support. In 2021, 56% of Portuguese in the city of Toronto were married or living common-law, with very few being divorced (4%) or separated (1.5%) (Statistics Canada, 2024).

Gentrification is transforming Little Portugal, prompting families to move to suburbs such as Mississauga and Brampton, seeking detached homes, green spaces, and family-friendly environments (Tardif-Grenier et al., 2023). Such pressures not only displace families geographically but also recalibrate how individuals perceive belonging, continuity, and the stability of their cultural reference points. Many Portuguese and Azoreans feel their culture is in transition. However, little is known about how the newer and young generations of Portuguese and Azoreans in Toronto relate to their parents’ heritage. Do they see the Portuguese-Azorean community as a reference point? How significant are its cultural and social institutions to them? Will they integrate into Canadian society, losing ties to their ancestral homeland, or remain caught between cultures?

Language retention is key to maintaining ethnocultural identity, yet Portuguese language use is steadily declining, especially in smaller communities. Language is also highly correlated to ethnic identity and may affect communication within multi-generational households (A. Oliveira & Teixeira, 2004; Vieira & Teixeira, 2023). This linguistic erosion is not merely a collective phenomenon; it also reshapes individual experience, as many younger Portuguese-Azoreans report a diminished sense of familial connection and cultural continuity when they no longer command their parents’ heritage language. Of the 448,305 Portuguese of ethnic origin living in Canada, only 53.7% declared Portuguese as their mother tongue, while 46.3% of second- and third-generation Portuguese reported having no knowledge of it (Statistics Canada, 2022, 2024).

In Canada, the Portuguese language is in a transitional phase, with new generations (second and third) using their parents’ mother tongue at home less frequently and preferring English when in public. The language usage is connected directly to affective patterns such as sense of belonging and motivation to engage with the main communities (Figueiredo et al., 2019; Figueiredo, 2008). Ontario has the largest population of Portuguese mother-tongue speakers (153,750 or 63.9%), most of them in Toronto, where 60,360 of 85,165 residents of Portuguese origin (60,360, or 70.9%) reported Portuguese as their first language (Statistics Canada, 2022, 2024). In Toronto’s suburbs and in smaller communities within the Toronto Census Metropolitan Area (excluding the city itself), in contrast, only 43.8% of those of Portuguese ethnic origin reported Portuguese as their mother tongue (Statistics Canada, 2022). This suggests a more complete level of cultural assimilation among “new” generations (second and third). Within this group, the linguistic duality that frequently characterizes the generation gap in immigrant households—where the children speak one language and the parents another—is becoming increasingly prevalent (DeSouza et al., 2023; G. Oliveira, 2021; A. Oliveira & Teixeira, 2004). Many Portuguese and Azorean youth engage in “code switching”—where English and Portuguese are mixed within a single conversation (Figueiredo & Silva, 2009; Figueiredo, 2019, 2023b; Vieira, 2021). Otherwise, the language is the main instrument to deal with psychological disturbances these immigrants experience, mainly adults (Figueiredo, 2023a; Gaspar da Silva, 2023). The gradual loss of their home language and culture has left some Portuguese and Azorean youth feeling isolated from their parents and alienated from the Portuguese-Azorean community as a whole. Younger generations are less likely to consume Portuguese media (TV, radio, and newspapers), and few participate in social and cultural events or give preference to Portuguese businesses and services (Figueiredo et al., 2016; A. Oliveira & Teixeira, 2004). Similar generational differences in language use have been reported by other immigrant groups and their descendants in North American cities and suburbs (Figueiredo & Henriques, 2024; Zhou & Bankston, 2020).

Toronto’s Portuguese-Azorean community nevertheless strives to maintain its language and culture as well as a strong cultural presence in “Little Portugal” and through numerous cultural, religious, and business institutions. In 2007, Ontario had 100 Portuguese-Azorean clubs, 40 language schools, 26 churches with Portuguese services, and around 4200 businesses. A decade later, Vieira (2021) identified about 100 cultural and social organizations in the Greater Toronto Area, many of which played vital roles in immigrant settlement and integration between the 1950s and the 1970s. Some continue to help community members by helping them find housing and employment. For many community members, these institutions act as anchors of resilience, enabling individuals and groups to sustain a coherent sense of identity amid broader assimilative pressures. While multiculturalism policies have encouraged the preservation of Portuguese culture, the next generation appears less engaged with these community institutions (Figueiredo et al., 2016; Vieira, 2021; Vieira & Teixeira, 2023). Canada’s state-sponsored multiculturalism policy may have served to encourage Portuguese immigrants to create ethnic-oriented organizations and to preserve their culture (Bloemraad, 2006; Qadeer, 2016). The latter organizations not only preserve and promote Portuguese language and culture for younger generations, they promote friendship and solidarity through a range of social, cultural, and recreational activities and function as a bridge between Portuguese-Azorean and mainstream Canadian culture. While their role and effectiveness have been the subject of debate over the last three decades (Da Rosa & Teixeira, 2009; Vieira & Teixeira, 2023) ethno-cultural organizations and multiculturalism have played a positive role in fostering tolerance and mutual respect among immigrant communities and cultures, including the Portuguese-Azoreans. For many immigrant groups, including the Portuguese it is expected that appeal of ethnic organizations will diminish overtime, especially among the more educated immigrants or new generations born in the host country. Portuguese youth in Canada often identify as “tricultural,” balancing Portuguese, Azorean, and Canadian influences. A study of 354 youth in Toronto and Montreal found that navigating multiple identities can be challenging (A. Oliveira & Teixeira, 2004). The future of Portuguese-Azorean identity in Canada thus remains uncertain.

## 4. Conclusions and Future Avenues of Research

What does the future hold for Portuguese and Azoreans, including the new and young generations in Toronto? In the second half of the twentieth century, Toronto has been transformed by a number of societal forces: economic restructuring, an ageing population, new approaches to family organization, and changes in immigration patterns. The Portuguese-Azorean community has demonstrated resilience and has adapted relatively well to its adoptive society. Portuguese-Azorean immigrants have adopted different settlement and residential patterns—from pockets of ethnic concentration marked by a rich and distinctive urban ethnic landscape and cultural identity (particularly among the first generation born outside Canada) to dispersion (mainly among second and third generations born in Canada) in the suburbs and “exurbs” of Toronto, including schooling in this ecological system.

Portuguese-Azoreans have only been in Canada for seven decades. With varying degrees of loyalty to their Portuguese and Azorean cultural heritage, the majority of this community’s members (first generation) came to stay. As the newer generations in Ontario attain maturity, levels of education are rising, and assimilation is expected to increase. While ethnic identity is a source of enrichment for some Portuguese-Azoreans, for younger immigrants it can result in conflict, and the rejection of their cultural heritage. It remains uncertain whether second- and third-generation Portuguese-Azoreans will preserve their parents’ traditions. Current evidence points to “cultural duality” and “cultural conflict” among youth, and whether this tension can be reconciled in the future is unclear. The role of Portuguese and Azorean immigrants and their descendants as agents of change in the social, cultural, political, and economic life of Canada and the city of Toronto, where most settled and raised their families, cannot be overstated.

The rich ethnic landscapes that Portuguese-Azoreans built in Toronto and other cities are testimony to their resilience and wish to preserve their heritage and culture on Canadian soil and highlight the evolving nature of Portuguese-Canadian identity. Given economic globalization, intense redevelopment pressures and Canada’s ‘demographic revolution’, the question is for how long will these built ethnic landscapes which offer space for everyday activities as well as staging of cultural heritage and social–cultural events will survive in the future? With that said, Portuguese and Azorean immigration has decreased drastically in the last three decades and Canada’s Portuguese-Azorean communities are currently in transition. The generations of Portuguese and Azoreans born in Canada must navigate the coming decades within diverse demographic, social, cultural, political, and economic contexts. There are still many questions about how the next generations will reinterpret these ethnic spaces, and how they will preserve or redefine their heritage for the future. This explains the ‘chronic urban trauma’.

It is appropriate to call for increased research and scholarly work on the Portuguese and Azorean communities in Canada, their “pioneers,” and descendants born in the country. Comparative scholarly work on the rich history of their migratory trajectories, settlement experiences, community formation, integration experiences—including cultural and mother-tongue retention, the “ageing” of their communities as well as their “ethnic imprint” on cities and local communities—remains limited. Given the importance of immigration to Canadian social and economic development, immigrant integration has become an issue of concern for Canadian academics and policymakers. Given evidence of rising stress in our cities—from immigrants at risk of exclusion, marginalization, urban poverty, social alienation, or even homelessness to racial tensions—it is crucial that the roles of ethnicity and race be addressed in future studies of Canadian immigrants.

In the ‘age of migration’ the adaptations processes and outcomes of immigrants have become more complicated. How immigrants shape the structure of cities and how these cities, in turn accommodate these immigrants’ culturally diverse needs and preferences is an increasingly important topic. Understanding the development of urban enclaves and ethnic dynamics on the neighbourhood scale is becoming increasingly critical as North American communities attempt to successfully integrate immigrants from very different cultures. Research into these relationships must get to the crux not only of immigrant settlement preferences, housing patterns, and work locations, but also of culture, religion, and leisure activities. It is crucial that scholars and policymakers better understand the complexities of immigrants’ integration “trajectories” into their new society, especially focusing young ages and schooling. In this context, its dynamic and prosperous Portuguese-Azorean community has contributed to Toronto’s current reputation as a multicultural “city of homelands” and bears witness to the power of immigration as an engine of demographic and economic growth and social as well educational transformation.

## Data Availability

The original contributions presented in this study are included in the article. Further inquiries can be directed to the corresponding author: Carlos Teixeira, Geography/Community, Culture and Global Studies, University of British Columbia, Kelowna, BC V1V 1V7.
